# Fatal Fungicide-Associated Triazole-Resistant *Aspergillus fumigatus* Infection, Pennsylvania, USA

**DOI:** 10.3201/eid2809.220517

**Published:** 2022-09

**Authors:** Kennedy Bradley, Audrey Le-Mahajan, Beth Morris, Tiina Peritz, Tom Chiller, Kaitlin Forsberg, Natalie S. Nunnally, Shawn R. Lockhart, Jeremy A.W. Gold, Jane M. Gould

**Affiliations:** Department of Public Health, Philadelphia, Pennsylvania, USA (K. Bradley, B. Morris, T. Peritz, J.M. Gould);; Hospital of the University of Pennsylvania, Philadelphia (A. Le-Mahajan);; Centers for Disease Control and Prevention, Atlanta, Georgia, USA (T. Chiller, K. Forsberg, N.S. Nunnally, S.R. Lockhart, J.A.W. Gold)

**Keywords:** Fungi, antimicrobial resistance, respiratory infections, *Aspergillus fumigatus*, antifungal drug resistance, invasive mold infection, triazoles, agriculture, Pennsylvania, United States

## Abstract

We report a fatal infection in a 65-year-old immunocompromised male patient caused by pan-triazole–resistant *Aspergillus fumigatus* containing a TR_34_/L98H genetic mutation linked to agricultural fungicide use. Clinical and environmental surveillance of triazole-resistant *A. fumigatus* is needed in the United States to prevent spread and guide healthcare and agricultural practices.

*Aspergillus fumigatus* is the most common cause of invasive aspergillosis, a life-threatening fungal infection that primarily affects immunocompromised persons, including those with hematologic malignancies or stem cell or solid organ transplants or those receiving immunosuppressive medications (*1*). Patients are infected by inhaling *A. fumigatus* spores found in the environment. Each year, invasive aspergillosis accounts for >14,000 hospitalizations and imposes >$1.2 billion in direct costs on the US healthcare system (*2*).

Voriconazole belongs to the triazole class of antifungal drugs and is a first-line treatment for invasive aspergillosis (*1*). Triazole drugs have improved patient survival; however, the emergence of triazole-resistant *A. fumigatus* threatens the effectiveness of triazoles in clinical practice (*3*). Patients with invasive aspergillosis caused by voriconazole-resistant *A. fumigatus* had a mortality rate of ≈60%, which was ≈2 times the mortality rate associated with voriconazole-susceptible infection (*4*). Patients can acquire triazole-resistant *A. fumigatus* infections because of exposure to long-term triazole therapy for chronic aspergillosis or by directly inhaling environmental spores that are already triazole-resistant (*3*). The agricultural use of triazole fungicides, a practice that recently increased 4-fold in the United States, can select for *A. fumigatus* strains harboring unique *CYP51A* gene mutations, such as TR_34_/L98H and TR_46_/Y121F/T289A, that can cause pan-triazole resistance in patients (*5*,*6*).

Reports of environmentally acquired triazole-resistant *A. fumigatus* infections are increasing worldwide; however, data on these infections and their clinical implications are lacking in the United States (*3*). We report a patient who died from an invasive infection caused by a pan-triazole–resistant *A. fumigatus* strain containing an environmentally acquired TR_34_/L98H mutation in *CYP51A*.

The male patient was 65 years of age and previously underwent chimeric antigen receptor T-cell therapy for acute myeloid leukemia. One month before hospital admission, the patient received an allogeneic stem cell transplant that was complicated by cutaneous graft-versus-host disease. Despite topical therapy, he was admitted to the hospital because of worsening rashes, fever, and lethargy. The patient received broad-spectrum antibacterial drugs and systemic corticosteroid therapy for progressive graft-versus-host disease involving the gastrointestinal tract and eyes and continued receiving transplant-related fluconazole prophylaxis.

On hospital day 3, the patient was transferred to the intensive care unit for wound management and treated for hypovolemic shock; his antifungal prophylaxis was changed from fluconazole to posaconazole. After 6 days, posaconazole was replaced with caspofungin because the posaconazole was potentially exacerbating the patient’s rash. The patient improved and remained hemodynamically stable for ≈2 weeks, after which clinicians deescalated antibacterial therapy.

On hospital day 23, acute-onset shock and hypoxemic respiratory failure developed in the patient; he was intubated and placed on mechanical ventilation. Chest computed tomography imaging showed multifocal pneumonia; bronchial cultures were positive for *A. fumigatus*. Clinicians initiated voriconazole therapy for probable invasive aspergillosis and continued caspofungin. On hospital day 27, progressive acidemia, refractory hypotension, and focal neurologic deficits developed in the patient. *Rhizopus* spp. was identified from the patient’s skin culture, but the patient was not treated for this pathogen because his family had decided to focus on comfort care. The patient died on hospital day 28. An autopsy determined that the cause of death was sepsis from disseminated *A. fumigatus* and *Rhizopus* spp. infections.

Although most US clinical laboratories do not perform antifungal susceptibility testing, triazole susceptibility testing for *A. fumigatus* isolates is available through the Centers for Disease Control and Prevention (CDC) Antibiotic Resistance Laboratory Network (https://www.cdc.gov/drugresistance/laboratories.html). Clinicians sent an isolate from the patient’s bronchial washings to CDC as part of an ongoing passive surveillance for triazole-resistant *A. fumigatus*. Using previously described methods (*7*), CDC performed broth microdilution to determine the MICs of itraconazole (>16 µg/mL) and voriconazole (2 µg/mL) for the isolate. The isolate was classified as voriconazole-resistant in accordance with Clinical and Laboratory Standards Institute MIC breakpoints (*8*). The MIC of itraconazole for the isolate was considered non–wild-type on the basis of proposed epidemiologic cutoff values (*9*). CDC performed DNA sequence analysis of the *CYP51A* gene and determined that the isolate contained the TR_34_/L98H mutation (*7*).

In summary, we report a fatal disseminated fungal infection in an immunocompromised patient in the United States involving pan-triazole–resistant *A. fumigatus* with an environmentally acquired TR_34_/L98H mutation. This report underscores the potential severity of triazole-resistant *A. fumigatus* infections in immunocompromised persons. Furthermore, clinicians should consider the possible presence of drug-resistant *A. fumigatus* in patients with invasive aspergillosis who do not improve with first-line therapy. In Europe, the emergence of environmentally acquired triazole resistance is well documented, and voriconazole monotherapy is no longer recommended as a first-line invasive aspergillosis treatment for patients in regions with environmental resistance rates of >10% (*10*). In the United States, systematic clinical and environmental surveillance for triazole-resistant *A. fumigatus* is needed to determine the spread of this fungus and guide clinical and agricultural practices.
